# Action Ethnography of Community Reintegration for Veterans and Military Service Members With Traumatic Brain Injury: Protocol for a Mixed Methods Study

**DOI:** 10.2196/14170

**Published:** 2019-11-22

**Authors:** Christine Melillo, Kiersten Downs, Christina Dillahunt-Aspillaga, Jason Lind, Karen Besterman-Dahan, Bridget Hahm, Nicole Antinori, Christine Elnitsky, Angelle M Sander, Heather G Belanger, Peter Toyinbo, Gail Powell-Cope

**Affiliations:** 1 Research and Development Service James A Haley Veterans’ Hospital Veterans Health Administration Tampa, FL United States; 2 Rehabilitation and Mental Health Counseling Program College of Behavioral and Community Sciences University of South Florida Tampa, FL United States; 3 School of Nursing College of Health and Human Services University of North Carolina at Charlotte Charlotte, NC United States; 4 Brain Injury Research Center TIRR Memorial Hermann Houston, TX United States; 5 H Ben Taub Department of Physical Medicine and Rehabilitation Baylor College of Medicine Houston, TX United States; 6 Defense and Veterans Brain Injury Center US Special Operations Command Tampa, FL United States

**Keywords:** ethnography, veteran, traumatic brain injury, community, military, social networks

## Abstract

**Background:**

Numerous studies of community reintegration (CR) in traumatic brain injury (TBI) have been conducted in civilian populations, but research is limited in veteran and military service member populations. Little is known about how knowledge from civilian studies translates into veterans’ experiences and needs. The US Department of Veterans Health Administration (VHA) recognizes the distinctive health care needs of post-9/11 veteran and military service members, particularly with TBI, including the need to bridge health and rehabilitation-related services from acute care and inpatient settings to veteran and military service members’ homes and communities to facilitate CR.

**Objective:**

The goal of this study is to better understand the experiences of veterans with complicated mild, moderate, or severe TBI; their families; and CR workers as veterans and servicemembers transition to and sustain living in communities. This paper describes the rationale, design, and methods used to reach this goal.

**Methods:**

This five-year longitudinal mixed methods study uses both a community-engaged research (CEnR) approach and an ethnographic approach. The sample includes 30 veterans and service members with TBI, 13 family caregivers, 11 CR specialists, 16 key stakeholders, and 82 community events. Interviews and observations are coded and analyzed using hierarchical coding schemes and thematic analysis. Analyses include data from surveys, interviews, and participant observations. Content analysis is used to highlight the complex social context of reintegration and to triangulate quantitative data. Egocentric (personal) social network analysis is used to examine the support system a veteran or service member has in place to facilitate reintegration.

**Results:**

Study enrollment and data collection are completed. Data analyses are underway.

**Conclusions:**

The results of this study may provide a heightened understanding of environmental factors affecting CR in complicated mild, moderate, or severe TBI. Veteran, servicemember and family voices and insights provide VHA clinicians and policy makers with an ecological view of CR that is grounded in the life experiences of veterans, military service members, and families. The results of this study provide a roadmap for designing and testing interventions to maximize CR in a variety of domains. The longitudinal ethnographic approach allows for capturing detailed experiences within the naturalistic context. CEnR allows collaborative assessment of the social context of reintegration with community members.

**International Registered Report Identifier (IRRID):**

DERR1-10.2196/14170

## Introduction

### Community Reintegration

Leading disability and rehabilitation organizations, researchers and clinicians recognize the importance of community reintegration (CR) to the health, quality of life, and well-being of persons with disabilities [1-3]. CR is defined as “the assumption or resumption of culturally and developmentally appropriate social roles following disability” [1]. Key components of CR emphasized by researchers, clinicians, and consumers include independence in daily life, involvement in productive or meaningful activities, and engagement in satisfying social relationships [4,5].

The Veterans Health Administration (VHA) recognizes the importance of CR in relation to the overall health and well-being of veterans. VHA programs exist to support CR, including vocational rehabilitation [6], community partnerships, and educational benefits [7-9]. Many of these programs tend to be medically based and disparate from one VHA facility to another VHA facility. The limited scope of and variation among these programs motivated the development of this study. The VHA is charged to effectively assist veterans with living healthy, meaningful, and productive lives. Neurological conditions, including traumatic brain injury (TBI), are leading causes of serious, long-term disability [10]. Since 2001, nearly 400,000 service members have been diagnosed with a brain injury [11], and more than 1.2 million newly separated veterans have accessed VHA health services [12]. In addition, complex comorbidities, such as mental health disorders, pain, and irritability, are likely to interfere with work, social functioning, and independent living and affect disability long-term CR outcomes [13,14]. Despite the heightened attention on TBI due to recent conflicts (Operation Enduring Freedom, Operation Iraqi Freedom, and Operation New Dawn [OEF/OIF/OND]) and associated deployments, stateside injuries account for the majority of severe TBIs in veteran and military service members [11]. These injuries pose unique challenges to the current generation of veterans returning to prior relationships, living situations, work, and social roles [15,16]. Most research on CR for veterans and servicemembers has focused on return to work and family life, with little examination of the social, physical, and environmental factors that affect CR [17,18].

To address these research gaps, the VHA Rehabilitation Research and Development Service convened a state-of-the-art conference in 2010 to advance the science of measurement for important outcomes for rehabilitation. One workgroup reported on directions for conceptualization and measurement of CR [5]. The workgroup acknowledged that the concepts of activity and participation in life roles (International Classification of Functioning concepts) and reintegration overlap and that CR is multidimensional and complex. The workgroup identified 11 key dimensions of CR for veterans with TBI, which were further distilled by Sanders to 3 dimensions of CR: (1) employment or other productive activity, (2) independent living, and (3) social relationships and activities [17]. In this study, we use Sander’s 3 dimensions as a framework for examining CR in this population [17].

Few studies to date have examined the subjective CR experiences of injured post-9/11 V, SMs and their family members [16,19,20]. Significant gaps in evidence of reintegration among veterans and servicemembers with specific health conditions or effective models of service delivery are identified [16]. Furthermore, there is a need to focus research on the intersection of individual, family, and community perspectives to adequately account for the complex context of reintegration. Experiences regarding barriers and facilitators to community resources and services are needed to inform VHA policy and service delivery models.

This research provides current and in-depth knowledge about CR in veterans and servicemembers with TBI from recent conflicts. This study uses a longitudinal design with multiple data collection methods from multiple stakeholder perspectives. The primary objectives of this research are to (1) describe CR experiences as perceived by veterans and servicemembers; (2) compare and contrast barriers and facilitators to CR from multiple perspectives (eg, veterans and servicemembers, families, and providers of CR-related health care and services); (3) describe how personal social networks influence CR; and (4) identify veterans and servicemembers patient-centered strategies needed to improve CR. This paper describes a protocol employing ethnographic research to investigate the experiences of veterans and servicemembers living with a TBI and their caregivers in the context of CR.

### Background

CR is an ongoing, complex process [14]. Consideration of individual perspectives helps shape the understanding of the complex interactions between outcomes and associated environmental CR factors [21]. Studying CR through multiple lenses facilitates capturing the rich, layered experiences of participants and addresses the multidimensional nature of reintegration from an intersectional perspective [16]. Study designs and approaches (eg, mixed methods, CEnR, and ethnography) are used to acknowledge this complexity.

### Theoretical Background and Ethnographic Approaches in Community Reintegration

Ethnography is the art and science used to describe a group or culture [22]. We use an ethnographic approach to learning about the social and cultural life of communities, institutions, and other settings [23]. Ethnographers use a variety of research methods and rigorous data collection techniques such as interviews, surveys, and participant observation to avoid bias and ensure accuracy of data collection and analyses [24]. This approach employs both inductive and deductive techniques to build culturally valid theories situated in local contexts. Ethnography builds on perspectives of the people situated within settings of interest [23]. The ethnographic approach in this protocol is used as a way to witness events in our participants’ world that may be beyond the reach of research approaches that are of more clinical nature [24].

The success of an ethnographic approach is dependent on rapport between participants and researcher [25]. Common techniques used in ethnographies to build a sense of rapport include participant observations and ethnographic interviews [25]. Participant observation involves detailed observation and recording of information about peoples’ lives. Ethnographic interviews occur over time, which helps to create a more complete picture of participant experience. This inductive, participant-perceived, and holistic approach of ethnography is ideal for studying veterans and servicemembers with TBI because it allows a diversity of perspectives to be represented.

There is a continuum of approaches to engage the community of interest. Community-engaged research (CEnR) relies on collaborative relationships between communities and researchers and is based on the understanding that communities best know the needs and concerns of their members [26]. This study uses CEnR as a collaborative research approach where the community informs the research process. Specifically, we relied heavily on the CEnR approach to share results of analysis with our community partners to facilitate interpretation and validation of concurrent study findings. Likewise, the community provides opportunities to translate findings into meaningful practice for community members and VHA alike. This approach to research involves all partners in the process and recognizes the unique strengths that each brings [27].

## Methods

### Design and Overview

**Figure 1:**
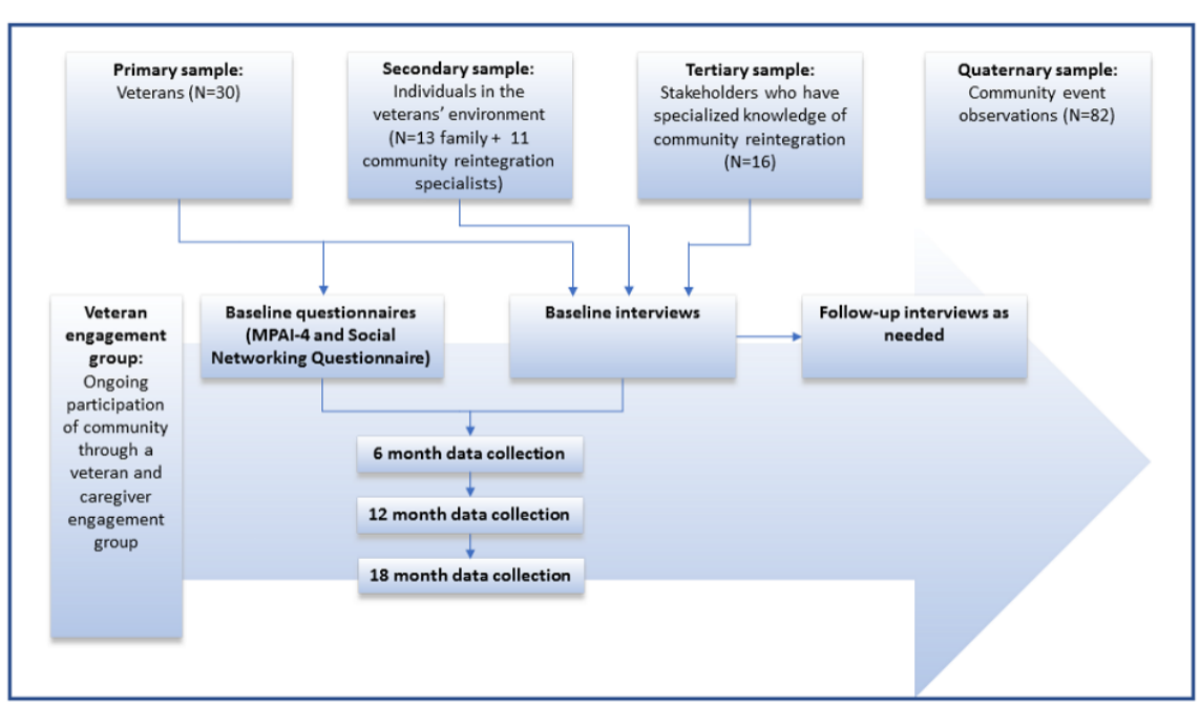
Schematic of study design data collection. MPAI-4: Mayo-Portland Adaptability Inventory-4.

This 5-year longitudinal ethnography study uses mixed methods (interviews, questionnaires, and participant observation) from a CEnR perspective (Figure 1). The combination of mixed methods is advantageous as it can provide (1) data and methodological triangulation, (2) a more complete understanding of research problems than either method on its own, (3) strengths that offset the respective weaknesses of both quantitative and qualitative research, and (4) answers questions that cannot be answered by either method alone [28]. Quantitative and qualitative methods can be combined in various ways depending on the timing of the collection of data, the relative weight of each, and how data will be used in analysis. A convergent mixed method design is used in this study [29]. Integration of findings from quantitative and qualitative components occur at the data analysis and interpretation phases.

Triangulation increases the validity, strength, and interpretative potential of the study; decreases investigator biases; and provides multiple perspectives of a given topic [30]. In this study, we employ a triangulation protocol as a strategy to integrate findings from different data sources. Specifically, convergent coding matrices [29] are used to achieve 2 types of methodological triangulation: (1) within-method triangulation (observation and interviews) and (2) across-method triangulation (structured questionnaires, interviews, and participant observations). The convergent coding matrix is used to display qualitative and quantitative findings that emerge in one place. This approach enables consideration of agreement, partial agreement, or dissonance between findings from different data sources [29]. The triangulation protocol facilitates the identification of meta-themes that cut across finding from different methods. After each qualitative data collection point, corresponding data are analyzed, and the results inform subsequent qualitative data collection for further clarification and probing of qualitative findings. When appropriate, triangulation occurs on both the individual and aggregate levels. Qualitative interviews, community observations, and CR surveys are compared and contrasted to determine the extent to which the data triangulate or converge [28]. Qualitative interviews from all 3 participant groups are compared and contrasted. Qualitative interviews and CR surveys are being triangulated with the social network questionnaires.

The collaborative nature of this study is an iterative process. The study incorporates a formal engagement of the target community through the veteran engagement group (VEG). Community engagement exists on a continuum of *“community*” (eg, immediate family, hospital unit, neighborhood, and city) and “*engagemen*t” (eg, transactional, transitional, and transformative) [31]. The VEG engages the community at large, building relationships and transforming the research process, as well as the community at large through information and connection.

### Ethical Review and Considerations

This study protocol was reviewed and approved by the affiliated university institutional review board as well as the local Veterans Affairs Research and Development Committee. These oversight entities look specifically for ethical considerations within each submitted protocol. The entities include attorneys, nurses, physicians, and veterans, among others, with expertise in evaluating safety and ethics of research protocols. Given the target population for the study protocol, persons diagnosed with complicated mild, moderate, and severe TBI, we used several measures to assure the protection of participants who consent to participate in the study, some of which are described in further detail in other parts of the protocol. Study staff underwent training and created study-specific procedures for working with participants who might voice thoughts of dying by suicide. Proxy consent and assent to participate were obtained for participants who were not competent to consent themselves [32]. Ongoing communications such as letters sent to participants include more information than would be used in populations without the potential for memory problems. In addition, team meetings often discussed the appropriateness of using data collection tools with this population.

### Sampling

#### Primary Sample

This sample includes veterans and servicemembers from OEF/OIF/OND with complicated mild, moderate, or severe TBI who received acute or transitional rehabilitation. Acute rehabilitation occurs immediately after veterans and servicemembers is medically stable and able to participate in rehabilitation. Transitional rehabilitation typically occurs after acute rehabilitation. Length of stay varies but is often a few months. veterans and servicemembers live in an apartment-style rehabilitation facility with a focus on functional, cognitive, and social goals. Length of stay is typically 1 to 6 months. Patients who have an anticipated or actual discharge from an acute or transitional care rehabilitation program, who are English speaking, and who have access to the internet are included. Patients who have severe substance abuse or severe disruptive behavior that could endanger participants or others, including data collectors, and anticipated discharge to or residence in an institutional facility are excluded. Proxy consent (from the legally authorized representative) is sought for those individuals determined not to be competent based on documentation in the electronic health record. Each veteran and servicemember remains active in the study for approximately 18 months to complete all data collection points.

Initially, the study design called for enrolling patients within 2 months of discharge from the acute or transitional rehabilitation programs. However, admissions of patients with moderate to severe TBI to the acute and transitional rehabilitation programs declined upon the inception of the study, resulting in very few patients eligible to participate in the study. In consultation with clinical coinvestigators and consultants, the study team expanded the inclusion criteria to include patients discharged within the past 12 months from acute or transitional rehabilitation. Ultimately, the decision was made to remove the time since discharge from acute or transitional rehabilitation criterion due to the difficulties stated above.

#### Secondary Sample

This sample consists of people identified by veterans and servicemembers as being within their social network and providing some level of support such as helping them at home or in the community and being important in their CR process. veterans and servicemembers identify potential participants for this secondary sample and provide contact information. For the purposes of this study, family is defined broadly as a person who provides a substantial amount of support as defined by the veterans and servicemembers and may be from family of origin or family of choice. CR specialists are defined as professionals who have assisted the veterans and servicemembers in their recovery after TBI (eg, case managers and VHA military liaisons) and are identified specifically by the veterans and servicemembers as an important asset to their rehabilitation. People who have involvement in care of the index veteran or servicemember and who are English speaking are included. There are no exclusion criteria. Each secondary sample participant remains active in the study for approximately 18 months to complete data collection.

#### Tertiary Sample

This sample consists of key community stakeholders who exert influence on the veterans and servicemembers indirectly through professional activities or medical and nonmedical appointments such as directors of community agencies (eg, Team Red, White, and Blue), people who work in relevant agencies such as VHA, State of Florida Department of Veterans’ Affairs staff, or local or state rehabilitation organizations. Stakeholders who have specialized knowledge related to care of veterans and servicemembers with TBI who live in the community are included. There are no exclusion criteria.

#### Quaternary Sample

This sample includes public events and spaces that are relevant to CR for veterans and servicemembers such as job fairs, open houses for organizations who provide veterans’ services, public art displays, and artistic performances. The study team uses multiple sources to identify events and resources that would be readily available to veterans and servicemembers and their families following their discharge from acute or transitional rehabilitation. Sources include local hospital outreach calendars; social media sites such as Facebook, Twitter, and Instagram; event calendars and announcements listed through local newspaper and media outlets; and websites for organizations such as veterans service organizations, nonprofit organizations, local cultural and sporting events, and community-based organizations.

### Sampling Size

The primary sample includes 30 veterans and servicemembers with complicated mild, moderate, or severe TBI. The secondary sample includes 13 caregivers and 11 CR specialists. The tertiary sample includes 16 key stakeholders. Typically, health services research with veterans and servicemembers with complicated mild, moderate, or severe TBI is limited due to the difficulty in obtaining a statistically powered sample size [33]. This study includes a methodology that capitalizes on small sample sizes. Although generalizability in the statistical sense may not be possible with the small sample size, the external validity of qualitative research will ultimately be determined by the usefulness of the findings in i comparable settings with similar veterans and servicemembers [34]. In qualitative research, data saturation is achieved not based on sample size but rather on the depth and breadth of information obtained from participants related to the phenomena under study [30,35]. Evidence shows that 20-60 knowledgeable people are enough to uncover and understand the key themes in a study of lived experience [36].

Multiple methods are used to recruit veterans and servicemembers who received care from a VHA Polytrauma Rehabilitation Center, either acute or transitional rehabilitation programs for recruitment into the study. Study staff advertise the study in inpatient rehabilitation areas through placement of study brochures and posters and announcements in relevant local newsletters. In addition, clinical partners refer potentially eligible participants to the research study team for screening. Recruitment and enrollment occur both before and after discharge from the acute and transitional rehabilitation programs with baseline data collection initiating after discharge. The study team conducts an eligibility review before enrollment using electronic health records and an internal program evaluation database to determine if a potential participant meets inclusion and exclusion criteria. Potential participants are contacted via United States paper mail if discharged or approached in person if still on site. Follow-up contact to schedule interview and survey data collection visits are conducted by phone calls and US mail.

Snowball sampling, whereby participants refer other people for participation [36], is used to identify the secondary sample. Participants in the secondary sample are contacted after verbal permission from the veterans and servicemembers. The tertiary sample is identified by the study team based on their knowledge of organizations and services relevant to CR; some participants in this sample are referred by other study participants. Telephone-assisted enrollment includes a waiver of documentation of consent. All study participants receive a copy of the informed consent (IC) form and a 1-page summary of consent by US mail as part of the IC process. The quaternary sample is identified by routine reviews of CR-related event sources. All study team members participate in the selection of events by reviewing event information by email or team meeting and identification of the team member(s) who will observe the event.

### Measures

Table 1 summarizes variables by sample, data collection instrument, and timing of data collection variables.

**Table 1:** Variables and measures.

Variable and definition			Sample	Data collection instrument	Data collection timing
**Demographics**					
	**Veterans and service members**				
		Age, gender, military service history, date of injury, and employment status	Primary	Questionnaire	Baseline
	**Caregiver**				
		Relationship to veteran or service member, length of time in relationship and caregiving, age, gender, and employment status	Secondary	Questionnaire	Baseline
	**CR^a^ specialist and stakeholder**				
		occupation, length of time in current role, experience related to CR, or veterans and servicemembers’ health	Tertiary	Questionnaire	Baseline
**Community reintegration**					
		Participation in community life that encompasses: (1) employment or other productive activity, (2) independent living, and (3) social relationships and activities.	Primary	Interview guide; Mayo-Portland Adaptability Inventory-4 Participation Index	Baseline and 6, 12, and 18 months
			Secondary	Interview guide	Baseline and 6, 12, and 18 months
**Barriers and facilitators to community reintegration**					
		Personal, interpersonal, or environmental factors that prevent or support engagement in employment or other productive activity, independent living, or social relationships and activities	Primary	Interview guide and field notes	Baseline and 6, 12, and 18 months
			Secondary	Interview guide	Baseline and 6, 12, and 18 months
			Tertiary	Interviews	One time and as needed
			Quaternary	Participant observation field notes and document reviews	Monthly and as needed
**Strategies to improve CR**					
		Activities intentionally undertaken by study participants to remove barriers and promote CR	Primary and secondary	Interview guide	Baseline and 6, 12, and 18 months
			Tertiary	Interviews	One time and as needed
			Quaternary	Participant observation	Monthly and as needed
**Personal social networks**					
	Relationships (ties) between an individual and members of own immediate social environment who interact with one another, and provide tangible social support		Primary	Social network questionnaire	Baseline and 6, 12, and 18 months

^a^CR: community reintegration.

### Interview Guide

Investigators employ open-ended, broad interview questions to capture veterans and servicemembers’ explanatory models of TBI and perspectives of CR. The research team developed the interview guide based on CR literature and study questions and objectives. Questions are modified as needed based on ongoing data collection and analysis.

### Personal Social Network Questionnaire

Investigators collect personal social support network information through the Social Network Questionnaire designed by the investigators. Veterans and servicemembers are asked to identify up to 5 other persons who provide them with emotional support, small services, large services, financial aid, and companionship.

Veterans and servicemembers also provide information on each identified person such as demographics, how long they have known each other, how often they talk to each other, and how that person is connected to them (eg, spouse, parent, siblings, friends, commander, or counselor). To capture personal social support network cohesion, veterans and servicemembers describe the relative strength of the relationship between each pair of their network contacts as to whether the network alters are “strangers,” “are as close to each other as I am to them,” or are “neither of these.”

### The Mayo-Portland Adaptability Inventory-4, Participation Index

The Mayo-Portland Adaptability Inventory-4 (MPAI-4) consists of 9 items with a 5-point ordinal scale [37]. Raters choose responses that best describe the level at which the person being evaluated experiences problems. Items tap into initiating activities, social contacts with others, leisure and recreation, self-care, independence in living situation, transportation, paid and unpaid employment, and management of finances. Items are summed to yield an overall score. The MPAI-4 has satisfactory internal consistency (Cronbach alpha=.89) and good content validity [38]. Although this instrument does not specifically frame results to our study CR domains. This instrument is designed to capture posthospitalization experiences and can be completed by health care providers, people with TBI, and their significant others. Ratings by different groups can be combined to provide a more reliable assessment.

### Data Collection Procedures

Figure 1 shows the data collection procedures and schedule for all samples. Formal in-person or telephone interviews are conducted with veterans and servicemembers (or proxies) every 6 months. Participant observation is conducted at event locations identified by researchers and participants to help researchers better understand CR. A semistructured field note template is used to record observations, interactions, and context by the study team member as soon as possible after attending community events. Photographs are taken as needed to supplement field note data.

Interviews are conducted by telephone or in person at the discretion of the study participant. Follow-up interviews are informed by survey and prior interview data. Interviews are audio recorded with the subjects’ consent, and the study investigator documents field notes, noting contextual features of the interview that are not captured in the recording (eg, nonverbal communication and characteristics of the setting). The interviews take a semistructured approach in that they are conducted with a preformulated interview guide, but they allow for answers to those questions to be fully expanded at the discretion of the interviewer and interviewee and enhanced by probes [23]. A flexible and dynamic approach is employed that provides an opportunity to uncover participants’ perspectives, using a conversational style to promote a spontaneous flow of information [39].

Web-based survey questionnaires are password protected. Qualtrics, a secure, Web-based survey tool, is used to record questionnaire data [40]. If the participant is unable to complete the Web-based survey, the interviewer assists in person or via telephone interview. Letters are mailed instructing participants on how to log in and access the Web-based survey, and reminder letters are mailed to ensure completion.

The CEnR approach requires meaningful engagement with veterans and servicemembers and family stakeholders. After initial attempts to engage study participants in a Web-based bulletin board forum were unsuccessful, a VEG was developed as an alternative method for veterans and servicemembers and family stakeholder engagement to assist study team members throughout the research process. Recent recommendations by the Health Services Research and Development Service Veteran Engagement Workgroup Final Report [41] support establishing VEGs to meaningfully engage veterans and servicemembers and significant others into research endeavors, thus ensuring veterans and servicemembers voices are well represented. This study’s VEG comprised 6 active volunteers. Members of the engagement group include veterans with a TBI (n=3) and caregivers for veterans with a brain injury (n=3). Participants have used VHA rehabilitation services for themselves or their loved ones. The research team developed the description and recruitment process for VEG members. Interested persons are recruited via word of mouth and via the James A. Haley Veterans’ Hospital and Clinics social media outlets—Facebook and Twitter. Of note, VEG participants are not research subjects enrolled in the study, but they serve as consultants to the research team. The VEG meetings vary in frequency from quarterly earlier in the study to bimonthly, as there is more data to share and input to solicit. This partnership yields positive results by providing feedback (eg, interpretation guidance and member checking) on early qualitative data analysis, advice for participant recruitment, and dissemination of early products. For example, the VEG is instrumental in the development of a stakeholder-driven Tip-Sheet derived from interpretation of data from local community event observations. The VEG also provides critical feedback on funded proposals informed by results of this study (eg, Resource Facilitation [42]), provides input on priorities for dissemination of current research findings and future next steps.

### Data Analysis

#### Qualitative

Qualitative insights from data analysis are used to iteratively guide subsequent data collection (eg, choice of next subjects [theoretical sampling], modification of interview questions, and feedback from subjects on researcher interpretations of data and provisional results). All interview audio files, field notes, and documents are transcribed or scanned and stored on a secure VHA server with access only to the research team.

A computer-assisted analysis model known as Noticing things, Collecting things, and Thinking about things (NCT) is employed to analyze the data [43,44]. The analysis process can be linear, starting with noticing interesting things in the data, collecting them (eg, as codes), thinking about them, and then writing interesting insights. However, more often, qualitative analysis requires moving back and forth between noticing, collecting, and thinking about things, as shown by arrows in the middle of the figure (see Figure 1). The NCT model uses coding structures, memoing, process mapping, and diagramming to describe, categorize, and connect the data to determine common themes patterns and inconsistencies relating to participants’ experiences, perceptions, and opinions.

Interview transcripts are uploaded into the qualitative analysis software program ATLAS.ti v8.0 which is used to organize data and systematically develop a codebook of the interview transcripts that catalogs and defines codes and thematic categories. The qualitative team meets regularly to analyze the interview transcripts in the following way: (1) assign first-level structured codes to units of meaning, (2) synthesize codes into complex categories, (3) compare and contrast categories to identify relationships across categories, (4) group categories into a taxonomic structure that describes the dataset, and (5) link sections of text to the coding to identify salient quotes that illustrate the codes and constructs and that support coding decisions. Memoing or analytic writing is also performed at each step of the analysis to create a written record of the process and to develop conceptual ideas relating to the data.

**Figure 2:**
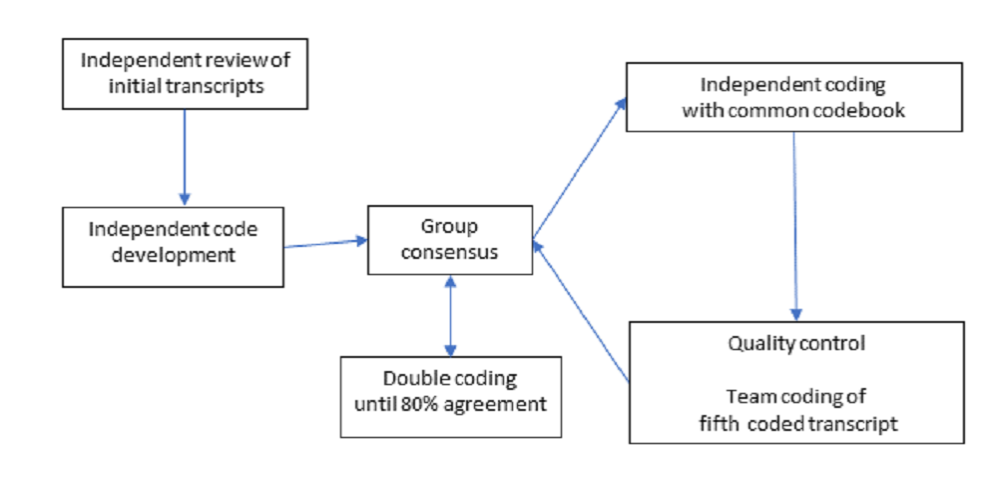
Coding process.

The qualitative team members compare and contrast perceptions of key findings following interviews. Investigators use the following analytic strategy: (1) reviewing the first few transcripts and developing codes independently; (2) reviewing their work together and, through consensus, agreeing on codes and definitions; (3) double coding transcripts until 80% agreement is attained; and (4) after 80% agreement is attained coding transcripts independently using the common codebook. Every fifth coded transcript is randomly reviewed by the team to select portions for agreement. Investigators revise and clarify the codebook as needed by discussing points of agreement and discrepancies and making decisions jointly to determine whether new codes were needed. Data analysts meet routinely to review ongoing coding results, resolve coding issues that arise, and discuss collapsing of codes into higher-level codes and constructs (Figure 2).

#### Quantitative

Egocentric social network analysis examines the presence of relationships between veterans’ personal (ego) social support network characteristics and successful CR as measured by the MPAI-4 [45]. Network visualization will be performed to investigate variables summarizing the number and types of ties in terms of the ego network’s shape, size, and composition and their changes over time. Regression analyses will identify which of these variables at baseline and/or over time show positive or negative effects on CR.

Veterans nominate persons of influence within their lives (alters). The connectedness among alters represents network density, and number of distinct alters represents network size. Interpretation of network size is deemphasized as by design, we limited the number of alters that an ego can name; however, up to this limit, the number of positive ties may positively influence reintegration. Higher density will suggest greater social cohesion, which is desirable. Measures of network composition will include the different types of connections between an ego and each alter and their frequencies (which denote connection strength), for example, same alter can be friend, coworker, and neighbor (multidimensional connection). The connection strength and/or type may be associated with CR. The network characteristics are collected for each ego network on each measurement occasion, which allows for observation of evolution of individual networks over time. The ego networks will be graphed, and the measures will be generated using E-NET software [46].

Using UCINET [47], CR at a given assessment (MPAI-4 scores as dependent variables) is modeled in a regression equation as a function of network-based mechanisms. Standard regression is used to analyze ego-level data (eg, density and demographics of ego). A 2-level regression model is fitted to alter-level data (eg, tie type, tie strength, and demographics of alter). Moreover, ego-level and alter-level data are tested for association with the rate of change of CR (linear slope) over time using 2-level and 3-level linear growth models, respectively.

## Results

The project was funded in FY2019 and enrolment was completed FY2019. Data analysis is currently under way and the first results are expected to be submitted for publication in 2020.

## Discussion

### Protocol Purpose

The goal of this study protocol is to describe methods used to better understand and highlight the experiences of veterans and servicemembers with complicated mild, moderate, or severe TBI; their families; and CR workers as they transition to and sustain living in communities. This protocol illustrates an ethnographic approach to support understanding of said experiences within the context of community. To our knowledge, this protocol is unique in that it explores CR over time from multiple perspectives, within the context of community.

### Strengths and Limitations

This protocol has several noteworthy strengths based on the CEnR approach. Specifically, the protocol was written to (1) provide a comprehensive understanding of veteran-perceived and contextual factors affecting CR to identify targets for interventions that can decrease barriers and strengthen facilitators for veterans with complicated mild, moderate, or severe TBI; (2) give voice to veterans and families by actively engaging them in removing barriers to CR directly for themselves and indirectly for others; and (3) provide a roadmap for designing and testing interventions to maximize CR in employment, independent living, and social relationships that are grounded in the perceptions of multiple perspectives.

Similarly, there are several limitations that are noteworthy of mention. First, the inclusion criteria originally included veterans and servicemembers who sustained a moderate or severe TBI and had recently been discharged from the local acute and transitional rehabilitation programs. However, because of decreasing military operations and changes in the patient populations being admitted to these programs, there were not sufficient numbers of eligible patients. To address this limitation, we expanded recruitment to those with complicated mild TBI and those who have received acute or transitional rehabilitation at any time. Second, the proposal originally planned to engage participants in the conduct of the study and analysis using a Web-based bulletin board for feedback and insights. This method proved ineffective, and the study team was not able to engage study participants in the Web-based bulletin board. However, the VEG provided an alternative method for engaging community-based participation in the research study. The purpose of this group is to provide a community for veterans and servicemembers and caregivers to pose questions and provide feedback on data and study issues as they arise. Finally, the study team planned on collecting quantitative measures of CR using both the Community Reintegration for Service Members (CRIS) 147 item measure and the participation index of MPAI-4. Although comprehensive, concerns about participant burden, and the ability of participants to complete the survey led the team to remove the CRIS and rely solely on the MPAI-4 participation index as no short-form scales were available for the CRIS. 

Finally, study data collection is limited to the Tampa Bay Area, which includes 1 of the 5 VHA polytrauma rehabilitation centers. The availability of community and VHA resources varies by region and county throughout the state of Florida. Greater resources are typically available in urban versus rural communities, affecting access to and use of services and supports. Access to Medicaid Waiver programs is also an important consideration. For example, the Traumatic Brain and Spinal Cord Injury Medicaid Waiver Program provides home- and community-based health care services for individuals aged 18 to 64 years with TBI or spinal cord injury in Florida. The waitlist for these services is long, and many individuals may never receive services through this program.

### Conclusions

This protocol employs an ethnographic mixed method design (interviews, observations, and surveys) and a CEnR approach. The intent is to elicit definitions and perceptions of CR and a more thorough understanding of environmental and cultural factors that influence CR in veterans and servicemembers with TBI. The addition of the VEG, which includes both veterans and servicemembers with TBI and caregivers of veterans and servicemembers with TBI, in the interpretation, feedback, and direction of findings; dissemination products; and next steps, supports meaningful engagement, input, and empowerment of the population being studied. Understanding of environmental and cultural factors that influence CR may inform VHA and Department of Defense policy and programmatic changes to support veterans and servicemembers as they transition to local communities and providers. The inductive, participant-perceived, and holistic approach of ethnography highlights the practical and participatory factors involved in understanding CR. Using this protocol, a comprehensive understanding of CR can be identified and disseminated, leveraging veteran and servicemember-focused experiences that can assuage CR barriers and strengthen CR facilitators for veterans and servicemembers with TBI. Future research should target development of programs and community collaborations to address CR barriers.

## Abbreviations

CEnR: community-engaged research
CR: community reintegration
CRIS: Community Reintegration for Service Members
IC: informed consent
MPAI: Mayo-Portland Adaptability Inventory-4
NCT: Noticing things, Collecting things, and Thinking about things
OEF: Operation Enduring Freedom
OIF: Operation Iraqi Freedom
OND: Operation New Dawn
TBI: traumatic brain injury
VHA: Veterans Health Administration
